# Evaluating quality of life and cost implications of prophylactic radiotherapy in mesothelioma: Health economic analysis of the SMART trial

**DOI:** 10.1371/journal.pone.0190257

**Published:** 2018-02-05

**Authors:** Samuel Alan Stewart, Amelia O. Clive, Nick A. Maskell, Erika Penz

**Affiliations:** 1 Dalhousie University, Halifax, NS, Canada; 2 Respiratory Research Unit, North Bristol NHS Trust, Bristol, United Kingdom; 3 Academic Respiratory Unit, School of Clinical Sciences, University of Bristol, Bristol, United Kingdom; 4 University of Saskatchewan, Saskatoon, SK, Canada; TNO, NETHERLANDS

## Abstract

**Background:**

The SMART trial is a UK-based, multicentre RCT comparing prophylactic radiotherapy and symptom-based (deferred) radiotherapy in 203 patients with Malignant Pleural Mesothelioma who had undergone large bore pleural interventions. Using costs and quality of life data collected alongside the clinical trial, we will estimate the cost-effectiveness of prophylactic radiotherapy compared to deferred radiotherapy over a 1-year period.

**Methods:**

Healthcare utilization and costs were captured during the trial. Utility weights produced by the EQ-5D questionnaire were used to determine quality-adjusted life-years (QALY) gained. The incremental cost-effectiveness ratio was calculated over the one-year trial period.

**Results:**

Costs were similar in the immediate and deferred radiotherapy groups: £5480.40 (SD = £7040; n = 102) and £5461.40 (SD = £7770; n = 101) respectively. There was also no difference in QALY: 0.498 (95% CI: [0.45, 0.547]) in the prophylactic radiotherapy group versus 0.525 (95% CI: [0.471, 0.580]) in the deferred group. At a willingness to pay threshold of £30,000/QALY there was only a 24% chance that prophylactic radiotherapy was cost-effective compared to deferred radiotherapy.

**Conclusions:**

There was no significant effect of prophylactic radiotherapy on quality of life in the intervention group, nor was there any discernable decrease in healthcare costs. There is little evidence to suggest that prophylactic radiotherapy is a cost-effective intervention in this population.

**Trial registration:**

ISRCTN72767336 with ISRCTN

## 1 Introduction

Malignant pleural mesothelioma is a cancer with a poor prognosis and few treatment options are available [1]. Patients often need multiple pleural interventions to diagnose and manage the tumor, which can result in the cancer spreading along the intervention tracts and “Procedure-Tract Metastases” (PTMs) developing [2]. Malignant pleural mesothelioma is sensitive to radiation therapy in vitro, but its use as radical treatment is precluded by the large doses that would need to be delivered to the other thoracic structures. However, radiotherapy (RT) can be safely delivered in smaller, prophylactic doses to intervention sites with minimal side effects [3, 4] and the efficacy of this has been evaluated in a number of randomised controlled trials [5–7].

Most recently, the SMART trial was designed to determine whether prophylactic RT would reduce the rate of PTMs in patients with malignant pleural mesothelioma undergoing large bore pleural procedures. 203 patients were randomized from 22 UK centres to receive either prophylactic RT or deferred RT (in the event a PTM developed). After the 12 months follow up period, 9 patients in the prophylactic RT and 16 patients in the deferred RT groups experienced a PTM (OR of 0.51, 95% CI: [0.19, 1.32], p = 0.14). The trial also showed no improvement in symptoms or quality of life in the prophylactic RT group and hence it was concluded that prophylactic RT was not an effective treatment in unselected patients with mesothelioma following pleural instrumentation [8].

Though prophylactic RT was not found to be statistically significant there were fewer PTMs in the prophylactic group. If the patients from the prophylactic RT group experienced lower costs then there might be an argument for pursuing further research into prophylactic RT. Using healthcare utilization and quality of life data that was collected throughout the SMART trial, we report a detailed analysis of the difference in healthcare costs and quality of life between the two groups and estimate the cost-effectiveness of prophylactic RT versus deferred RT in patients with malignant mesothelioma undergoing pleural interventions.

## 2 Methods

SMART was a multi-centre, open label, phase 3 randomized controlled trial that randomized 203 patients from 22 hospitals in the UK with histocytologically confirmed malignant pleural mesothelioma who had large-bore pleural interventions in the 35 days prior to recruitment [8]. Ethical and regulatory approval for the study was obtained from South Central (Southampton B) Ethics Committee of the UK National Research Ethics Service before recruitment commenced (REC number: 11/SC/0408). After written informed consent was obtained, patients were randomized to immediate (prophylactic) RT within 42 days of pleural intervention or deferred RT. Patients in the deferred group were only given RT after the occurrence of a PTM (i.e. were treated as usual for this patient population). Information related to the procedures have been described in detail previously [8]. All patients were followed monthly for 1 year by telephone and on a 3-month basis in person at a hospital clinic. The primary outcome of the trial was the incidence of PTM within 12 months from randomization (defined as a clinically palpable nodule at least 1cm in diameter within 7cm of the margin of the pleural intervention site). Secondary outcomes included chest pain visual analogue scores over 12 months, quality of life using the EQ-5D and QLQ-30 questionnaires, survival, adverse events and healthcare utilization and patient experience.

In this paper we conducted a cost-effectiveness analysis alongside the clinical trial from the perspective of the healthcare payer, therefore non-medical costs were not included in the analysis (i.e. patient time and travel costs, costs related to lost productivity, etc.). All patients were followed for 1 year or until death, whichever occurred first, and the analysis was performed over this time frame. Median survival was 357 days and 365 days in the immediate and deferred RT groups respectively, therefore modeling of costs beyond the trial period was not performed. Given that costs included in the analysis were incurred over the trial follow up period of up to 1 year, discounting was not performed.

### 2.1 Healthcare utilization and costs

Healthcare utilization was measured from the perspective of the healthcare payer, the UK National Health Service (NHS). Healthcare resource utilization was captured in person at months 1, 3, 6, 9 and 12 and over the telephone for the intervening months. The following healthcare system utilization variables were captured: A&E visit (manually or by ambulance), outpatient appointments and profession seen, hospital and ICU stays, radiation, chemotherapy, thoracic procedures, palliative care and pleural procedures received, along with what analgesia medications were received. Costs were taken from multiple sources, including the NHS Enhanced Tariff Option [9], the Personal Social Services Research Unit (PSSRU) Unit Costs of Health and Social Care 2014 [10], and other sources [4, 11]. Table 1 reports the specific costs for each procedure, and appendix A in S1 File details where each individual cost can be found. As some cost sources were from different years costs were inflated to 2015 and reported in 2015 UK pounds (£), see appendix A in S1 File for details.

**Table 1:** Healthcare utilization and costs summary. See appendix A in S1 File for a detailed description of cost sources.

	Prophylactic Radiotherapy		Deferred Radiotherapy		Cost Per Unit	Prophylactic Radiotherapy	Deferred Radiotherapy
	n	Mean (SD)	n	Mean (SD)		Cost, £ (SD)	Cost, £ (SD)
*Mean Total Cost per Patient*						5480.4 (7039.6)	5461.4 (7770)
*Emergency Visit-Ambulance*	25	0.2 (0.5)	36	0.4 (0.7)	£223	54.7 (110.7)	79.5 (162.6)
*Emergency Visit-Other*	50	0.5 (0.7)	65	0.6 (1)	£170	83.3 (121.4)	109.4 (164.1)
*Chemotherapy*	111	1.1 (1.2)	140	1.4 (1.2)	£300	324.3 (356.1)	413.1 (369.7)
*Chemo Dose*					£119	3.5 (20.2)	8.2 (42.1)
*Hospital*: *>1 Day*	73	0.7 (1.1)	79	0.8 (1)	£3,204	2293.1 (3503.4)	2506.1 (3317)
*Hospital*: *1 Day*	16	0.2 (0.5)	34	0.3 (0.6)	£961	150.7 (443.3)	323.5 (612)
*Hospital*: *Same Day*	26	0.3 (1.1)	27	0.3 (0.8)	£961	245 (1062.1)	256.9 (802.8)
*Days in Hospital*	730	7.2 (22.1)	664	6.6 (12.6)			
*Days in ICU*	59	0.6 (3.2)	54	0.5 (4)	£904	878.5 (4903.5)	812 (6031.3)
*Outpatient*: *Doctor*	446	4.4 (3.5)	467	4.6 (3.6)	£60–420	240.4 (235.1)	202.5 (186.5)
*Outpatient*: *Nurse*	145	1.4 (2.3)	155	1.5 (2.3)	£64	91 (149.5)	98.2 (145.3)
*Outpatient*: *Other*	177	1.7 (2.4)	72	0.7 (1.3)	£35	60.7 (83.6)	25 (44.1)
*Palliative Care*	114	1.1 (1.2)	102	1 (1.2)	£277	309.6 (331.4)	279.7 (335.8)
*Pleural Procedure*: *Any*	15	0.1 (0.5)	38	0.4 (0.9)			
*Pleural biopsy*	1	0 (0.1)	1	0 (0.1)	£176	2 (20)	2 (20.1)
*Indwelling Pleural Catheter*	1	0 (0.1)	3	0 (0.2)	£475	5.4 (54.1)	16.2 (93.2)
*Diagnostic pleural aspiration*	7	0.1 (0.3)	17	0.2 (0.4)	£176	13.9 (51.4)	34.1 (90.8)
*Chest drain Insertion*	2	0 (0.1)	4	0 (0.2)	£226	5.1 (36.2)	10.3 (50.9)
*Pleurodesis*	0	0 (0)	2	0 (0.1)	£268	0 (0)	6.1 (43.2)
*Therapeutic pleural aspiration*	4	0 (0.2)	10	0.1 (0.3)	£176	7.9 (39.5)	20 (60.8)
*Radiotherapy Received*	100	1 (0.4)	11	0.1 (0.3)	£277	271.6 (103)	30.2 (86.7)
*Cost Per Fraction*					£92	287.7 (171.1)	45.5 (166.4)
*Thoracic Surgery*	0	0 (0)	4	0 (0.2)	£1,990	0 (0)	78.8 (390.1)
*Medication Costs*					*	152.1 (266.7)	104 (182)

### 2.2 Utility scores and quality of life

Quality of life in the SMART trial was captured using the European Organization for Research and Treatment of Cancer Quality-of-Life Questionnaire—Core 30 (QLQ-C30) and the EuroQol-5D (EQ-5D). Patient responses to the EQ-5D were converted to utility scores, ranging from 0 (death) to 1.0 (perfect health), at each follow up period using the UK valuation set [12]. The EQ-5D survey can describe 243 unique health states, which can be valued in a single utility score between 0 and 1. In the UK, direct valuation of a subset of 42 health states were obtained from a sample of 3395 members of the public in 1993 [12]. A linear regression was then used to predict valuations of all other health states generated by the EQ5D survey. The regression coefficients for each level of each health state are reported in Appendix B in S1 File. Quality adjusted life years (QALYs) were determined by combining utility scores derived for each patient at baseline and each follow-up period (1, 3, 6, 9 and 12 months) using area under the curve methodology.

For patients with missing baseline utility scores, (4 patients, 98% completion) we imputed their baseline value using the mean utility score associated with their intervention group. For patients who survived the 12 months of the trial but missed their last assessment (16 patients, 85% completion) their last valid measurement was carried forward. For patients that withdrew (4 patients, 98% completion) their last recorded utility score was carried forward to their end of study (either death or 365 days after entry). If an interval measurement was missed (76 missed total, 97% completion rate) then no imputation is needed. For patients who died their utility score was set to zero at their date of death and carried forward until the end of study. Differences in baseline utility scores were controlled for using linear regression methods proposed by Manca, Hawkins and Sculpher [13].

### 2.3 Cost effectiveness analysis

To calculate the incremental mean cost and QALY difference between the two groups we used bootstrapping with 1000 replications to derive an estimate and 95% confidence interval. To check for potential effects of skewing in the costs we investigated both average and median cost and QALY per bootstrap sample. To assess the probability that an intervention was cost-effective at different willingness to pay thresholds for an additional quality adjusted life-year gained, cost effectiveness acceptability curves (CEACs) were used. Incremental cost-effectiveness was calculated as the ratio of the difference in costs between the intervention and control groups divided by the difference in QALYs gained between the groups. Incremental cost-effectiveness ratios (ICERs) were calculated for the trial period. Given that the ICERs could be negative (either the intervention was less expensive and more effective, or in contrast, less effective and more expensive), the ranking of these bootstrap estimates would potentially be misleading, so 95% confidence intervals were not presented. The use of the CEACs better illustrates the uncertainty of the baseline ICER estimate within the context of a funder’s willingness to pay.

Finally, to supplement the costs investigations we performed a secondary survival analysis using Kaplan Meier curves and Cox Proportional Hazards regression to evaluate if PTMs were associated with overall survival.

All analyses were performed in R version 3.2.2 (R Core Team, https://www.R-project.org/). Significance was set at α = 0.05 for the calculation of 95% confidence intervals and interpretation of statistical results.

## 3 Results

Table 2 presents the patient demographics, overall and by treatment group. No difference in baseline characteristics were seen between groups. Study participants were predominantly male (89%), with 65% of participants between the ages of 65 and 80.

**Table 2:** Patient demographics, disease and treatment information, overall and by intervention group.

Variable	Level	All (%)	Intervention (%)	Control (%)
Sex	Male	181 (89)	91 (89)	90 (89)
	Female	22 (11)	11 (11)	11 (11)
Age	<65	38 (19)	21 (21)	17 (17)
	65–80	133 (66)	66 (65)	67 (66)
	80+	32 (16)	15 (15)	17 (17)
Length of Follow-up from trial entry to death, withdrawal or 12 months.	12 months	105 (52)	52 (51)	53 (52)
	6–11 months	52 (26)	28 (27)	24 (24)
	<6 months	46 (23)	22 (22)	24 (24)
Died	No	98 (48)	48 (47)	50 (50)
	Yes	105 (52)	54 (53)	51 (50)
Type of pleural intervention	Large-bore chest drain insertion	3 (1)	1 (1)	2 (2)
	Local anaesthetic thoracoscopy	74 (36)	38 (37)	36 (36)
	Thoracotomy	9 (4)	3 (3)	6 (6)
	Video-assisted thoracoscopic surgery	91 (45)	45 (44)	46 (46)
	Indwelling pleural catheter insertion	25 (12)	14 (14)	11 (11)
	Other	1 (1)	1 (1)	0 (0)
Mesothelioma histological subtype	Epithelioid only	142 (70)	71 (70)	71 (70)
	Sarcomatoid	16 (8)	8 (8)	8 (8)
	Biphasic (mixed)	37 (18)	19 (19)	18 (18)
	Desmoplastic	4 (2)	4 (4)	0 (0)
	Other	4 (2)	0 (0)	4 (4)

No significant difference was seen in the overall healthcare utilization or health care costs between groups (Table 1 contains a summary of the healthcare utilization and healthcare costs). Mean cost over the trial period was £5480.40 (SD = 7040) in the prophylactic RT group versus £5461.40 (SD = 7770) in the deferred RT group. Accounting for survival over the 1 year follow up period, there was no difference in QALYs between groups, with a mean QALY of 0.498 (bootstrapped 95% CI: [0.450, 0.547]) in the prophylactic RT group and a mean QALY of 0.525 (95% CI: [0.471, 0.580]) in the deferred RT group.

Table 3 presents a summary of the mean costs and quality of life for both groups, stratified by presence/absence of PTMs. For patients with PTM, mean QALY was slightly higher and mean costs slightly lower, though the differences were minimal and not statistically significant.

**Table 3:** Summaries of the Quality of Life Years (QALY) and costs, both overall and stratified by the study outcome (occurrence of a procedure tract metastasis, or PTM).

	n	Mean	SD	Median	IQR
QALY	203	0.510	0.291	0.510	[0.24, 0.77]
QALY-PTM	25	0.518	0.286	0.594	[0.28, 0.73]
QALY-No PTM	178	0.509	0.293	0.500	[0.24, 0.77]
Costs	203	£5,470.93	£7,393.60	£3,669.93	[£1646.03, £6619.58]
Costs-PTM	25	£4,911.69	£3,392.73	£4,329.46	[£2008.80, £7064.56]
Costs-No PTM	178	£5,549.47	£7,795.85	£3,481.00	[£1546.73, £6493.79]

**Fig 1:**
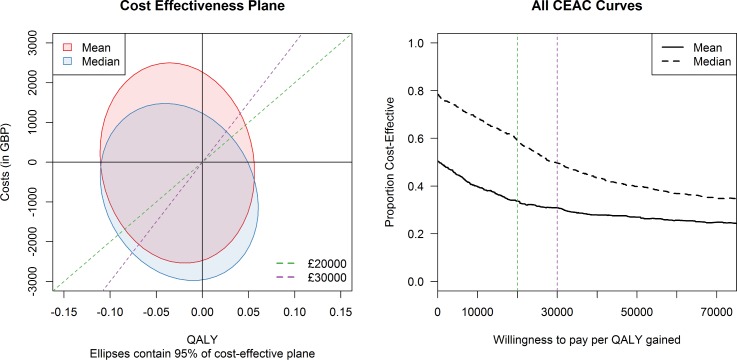
Cost effectiveness plane and CEAC for the intervention. The lines on the cost-effectiveness plane (left) represent willingness-to-pay thresholds of £20,000 (green) and £30,000 (purple). The same willingness to pay thresholds are marked with vertical dotted lines on the CEAC (right). The ellipses represent the area in which 95% of the bootstrap sample values fall.

Table 4 presents a summary of the incremental costs, QALYs and incremental cost-effectiveness ratio (ICER), using both the mean-cost and median-cost per bootstrap sample, while Fig 1 presents the Cost Effectiveness Plane and CEAC depicting the probability of prophylactic RT being cost-effective compared to deferred RT at various willingness to pay thresholds. At a willingness to pay threshold of £30,000/QALY, the probability that prophylactic RT is cost-effective compared to deferred RT is 31% using mean-cost analysis and 49% using median-cost analysis.

**Table 4:** Summary of the cost-effectiveness analysis, with incremental costs and QALYs, and the resultant ICER.

	Mean-Analysis		Median Analysis	
	Average	95% CI	Average	95% CI
*Prophylactic RT Cost*	£5,450.29	[£4270.35, £6834.93]	£3,082.82	[£2232.58, £4287.22]
*Deferred RT Cost*	£5,453.86	[£4172.01, £7035.46]	£3,831.52	[£2303.62, £4790.66]
*Cost Difference*	-£3.57	[£-2100.25, £1934.5]	-£748.71	[£-2170.78, £1380.43]
*Prophylactic RT QALY*	0.498	[0.45, 0.55]	0.496	[0.444, 0.548]
*Deferred RT QALY*	0.525	[0.47, 0.58]	0.522	[0.468, 0.578]
*QALY Difference*	-0.027	[-0.1, 0.04]	-0.02	[-0.095, 0.042]
*Incremental Cost-effectiveness ratio*	£132.04		£28606.46	

**Fig 2:**
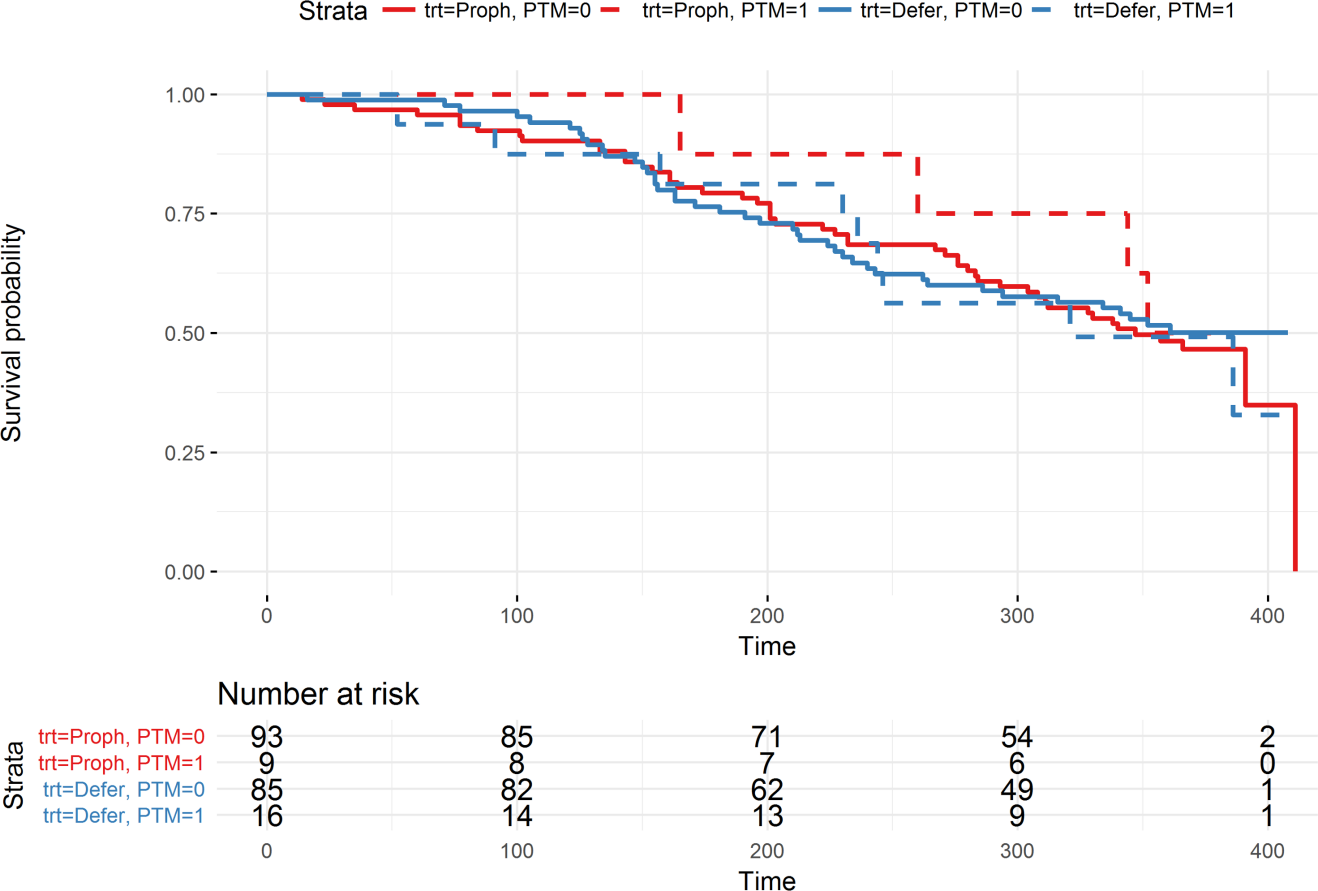
Kaplan Meier curves for treatment and PTM event. The survival rates for prophylactic treatment patients are presented in red, deferred treatment in blue. Patients that experienced a PTM are drawn with dashed lines, no-PTM patients with solid lines. At the bottom of the figure the number of patients at risk at each 100-day interval are noted. Some patients go beyond 365 days (study duration) due to the delay in scheduling their final appointment.

Finally, Fig 2 presents the survival curve for the patients, stratified by treatment group and PTM. A Cox Proportional Hazard regression model was built predicting survival using presence of a PTM and treatment group. The HR for patients that received deferred RT was 0.96 (95% CI: [0.7, 1.4]) compared to patients receiving the prophylactic RT, while the HR for patients experiencing a PTM was 0.99 (95% CI: [0.6, 1.8]) compared to patients not experiencing a PTM, suggesting that there was no effect of either the prophylactic RT or of experiencing a PTM on overall survival.

## 4 Discussion

Health economic analysis of the SMART trial has shown that from a healthcare payer perspective, prophylactic RT to pleural intervention sites in Mesothelioma is unlikely to be cost-effective compared to deferred RT delivered if a PTM develops. No clinically or statistically significant differences were identified in the mean costs, survival or quality of life of the two groups. The primary analysis of the SMART trial data [8] also failed to identify a difference in PTM rate between the treatment groups, concluding that delivery of prophylactic RT to unselected patients with mesothelioma can not be justified.

The concept of delivering prophylactic RT in mesothelioma assumes that it is effective at reducing PTMs and that its side effects outweigh the symptoms of a PTM. The SMART trial found that (a) prophylactic RT did not reduce the rate of PTMs significantly, and (b) that patients in the deferred RT group did not have a significantly lower quality of life. This health economic analysis has added to these conclusions by demonstrating that (i) there was no difference in the costs incurred between the two groups, and that (ii) patients that experience a PTM in either group did not have a noticeably lower quality of life score. If PTMs do not significantly influence quality of life or survival then it is difficult to justify treating them prophylactically, especially when prophylactic treatment does not completely reduce the risk of experiencing a PTM. Further data from the PIT trial (a UK based RCT evaluating prophylactic irradiation of tracts in mesothelioma) is awaited with interest as this will provide further efficacy and symptom control data, although no health economic analysis has been planned as part of this trial [14].

We have demonstrated that there is between a 31%-50% chance of prophylactic RT being cost effective at £30,000/QALY, the maximum value at which the National Institute of Health and Care Excellence (NICE) considers an intervention to be cost-effective [15]. As seen in Fig 1, when the willingness to pay increases the cost-effectiveness of prophylactic radiotherapy decreases. This is because, for most of the bootstrap samples, the prophylactic RT group had a slight decrease in quality of life overall with no decrease in costs. We would caution against trying to draw conclusions about the difference in quality of life between the prophylactic and deferred groups, the difference is small and the bootstrapped CI covers 0, suggesting that the quality of life is roughly the same between the two groups. The difference in median and mean estimates suggests that the data is slightly long-tailed, but further investigation into these high-cost patients did not suggest that they were outliers, and since costs incurred appeared to be relevant to either the disease or the intervention, they were not omitted from the analysis. We opted to present both the mean and median analysis in the paper as there is some discussion in the cost-effectiveness literature that both measures can be helpful in cost-effectiveness analysis interpretation [16]. Given their overall agreement, that the probability that prophylactic RT is cost effective is low, is helpful in reducing uncertainty about the value for money of the intervention for a decision maker.

Table 3 demonstrates two surprising findings: That patients diagnosed with a PTM had neither a noticeable drop in quality of life nor an increase in healthcare system costs. Our study was not sufficiently powered to explain these findings, but for the costs we note that the average cost for the prophylactic RT group was only about £480 more than the deferred group, and considering the size and variation of the costs overall in Table 1 the price of treating a PTM is quite minor. For the lack of difference in quality of life our only hypothesis is that, with a 50% mortality rate at 1 year, the effect of a PTM may not be as impactful on overall quality of life as was initially believed. This is not in anyway to suggest that PTMs should not be treated, rather that the process of diagnosis and treatment of PTM may not be sufficiently taxing on the patient to warrant prophylactic treatment.

The SMART trial has several strengths. It is a robustly conducted, large randomised controlled trial and is the largest, suitably powered study to evaluate the effects of prophylactic radiotherapy in mesothelioma. Unlike the previous RCTs in this field the symptoms and quality of lives of patients in the trial were carefully evaluated, which adds important data regarding these important patient-centered outcomes to the published literature. To our knowledge, this is the first time the health economics of prophylactic irradiation of tracts has been evaluated in this population. This analysis was planned a priori and published with the protocol prior to data analysis [17].

There are some limitations of our study. The time frame of the study was one year, and given that close to half the population was still alive at 1 year we may have missed important health outcomes beyond the scope of the trial. The analysis of a single payer system like the UK may make the generalizability to other countries more difficult, as the costs incorporated into our analysis may not be relevant to their systems. We have not included the costs of the chemotherapy drugs themselves to the analysis due to insufficient data regarding the specific drugs and doses administered, however the proportion of patients receiving chemotherapy in the two groups was comparable [8]. This was a secondary analysis of the SMART trial data and not necessarily powered to detect meaningful differences in healthcare costs.

## 5 Conclusion

Based on data collected alongside the SMART randomized clinical trial, it is unlikely that immediate prophylactic RT is cost-effective compared to deferred RT in preventing procedure tract metastases in patients with malignant mesothelioma undergoing pleural procedures. There was a no significant effect of prophylactic RT on quality of life in the intervention group, nor was there any discernable decrease in healthcare costs.

## Supporting information

S1 File (Appendix A) Source of Costs (Appendix B) Quality of Life Conversions.
(DOCX)
