# Supplementary material for: Evaluating quality of life and cost implications of prophylactic radiotherapy in mesothelioma: Health economic analysis of the SMART trial
Source: PLoS One. 2018 Feb 5;13(2):e0190257. doi: 10.1371/journal.pone.0190257 (PMC5798762; doi:10.1371/journal.pone.0190257)
Supplement: S1 File — (Appendix A) Source of Costs (Appendix B) Quality of Life Conversions. (DOCX) [file pone.0190257.s001.docx]

# Appendix A: Source of Costs

- The PSSRU Unit Costs of Health and Social Care 2014, compiled by Lesley Curtis [1] (<http://www.pssru.ac.uk/project-pages/unit-costs/2014/>). The section and page of the report used is provided for each resource.
- The National Health Service (NHS) Tariffs for 2015/16 [2] (<https://www.gov.uk/government/publications/tariff-arrangements-for-your-201516-nhs-activity>). The billing code(s) and names of billing codes are provided when used
- Two other papers are used that extracted similar information in other publications, and are listed below. For the Penz paper the costs were from 2011 so they were inflated by a factor of 1.15, and for the Edbrooke paper the costs were from 1996 so they were inflated by 1.68. Both inflation factors taken from <http://inflation.stephenmorley.org/>

|  | Cost | Source | Source |
| --- | --- | --- | --- |
| Emergency Visit-Ambulance | £223 | PSSRU | 8.1: NHS reference costs for hospital services; Ambulance Services; See and treat and convey |
| Emergency Visit-Other | £170 | NHS | Section 03. A&E |
| Chemotherapy | £300 | NHS | SB13Z: Deliver more complex Parenteral Chemotherapy at first attendance |
| Chemo Dose | £119 | NHS | SB11Z: Deliver exclusively Oral Chemotherapy |
| Hospital: >1 Day | £3,204 | NHS | DZ16A: Without documentation of reason for admission we used price for “Pleural Effusion with Major CC” as a default cost of stay. |
| Hospital: 1 Day | £961 |  |  |
| Hospital: Same Day | £961 |  |  |
| Days in ICU | £904 |  | Edbrooke et al [3] |
| Outpatient: Doctor | 60-420 | NHS | Section 02. When possible we used the appropriate specialist and first/follow-up code, when in doubt we defaulted to a follow-up radiologist visit |
| Outpatient: Nurse | £64 | PSSRU | 10.4: Nurse specialist in the community, 1 hour of patient-related work |
| Outpatient: Other | £35 | PSSRU | 13.5: Hospital radiographer, 1 hour of work. We assumed that patients in this study, if not seeing a doctor or nurse, were most likely to see a radiography-related technician. |
| Palliative Care | £277 | PSSRU | 7.1 NHS Reference costs for hospital services; Palliative Care |
| Pleural Procedure |  |  | Penz et al [4] |
| Biopsy | £176 |  | Penz et al [4] |
| Catheter | £475 |  | Penz et al [4] |
| Diagnostic | £176 |  | Penz et al [4] |
| Drain Insertion | £226 |  | Penz et al [4] |
| Pleuro | £268 |  | Penz et al [4] |
| Therapeutic | £176 |  | Penz et al [4] |
| Thoracic | £475 |  | Penz et al [4] |
| Radiation Received | £277 | NHS | SC47Z: Preparation for simple radiotherapy with imaging and simple calculation |
| Cost Per Fraction | £92 | NHS | SC22Z: Deliver a fraction of treatment on a megavoltage machine |
| Thoracic Surgery | £1,990 | NHS | Average of four codes:  DZ03A Major Thoracic Procedures with CC  DZ03B Major Thoracic Procedures without CC  DZ04A Intermediate Thoracic Procedures with CC  DZ04B Intermediate Thoracic Procedures without CC |
| Medication Costs | * | NHS | NHS Drug Tarrif Part VIIIA, using recorded patient information about medications |

[1] Curtis L. Unit Costs of Health and Social Care 2014. 2014. Personal Social Services Research Unit, University of Kent, Cantebury.

[2] NHS England. Guide to the Enhanced Tariff Option for 2015/16. 2015. <https://www.gov.uk/government/publications/tariff-arrangements-for-your-201516-nhs-activity>. Accessed 2017-06-08

[3] Edbrooke DL, Ridley SA, Hibbert CL, Corcoran M. Variations in expenditure between adult general intensive care units in the UK. Anaesthesia. 2001 Mar;56(3):208-16.

[4] Penz ED, Mishra EK, Davies HE, Manns BJ, Miller RF, Rahman NM. Comparing cost of indwelling pleural catheter vs talc pleurodesis for malignant pleural effusion. Chest. 2014 Oct;146(4):991-1000. doi: 10.1378/chest.13-2481.

# Appendix B: Quality of Life Conversions

The EQ-5D survey used for the SMART trial had 5-questions, each with 3 Likert-scale responses, as outlined below. A patient with full health has a utility score of 1, and for each 2 or 3 value recorded the corresponding coefficient value is deducted from their health score. In addition, any value of 2 or 3 results in a deduction of -0.081, and any value of 3 results in an additional deduction of -0.269. These coefficients were developed specifically for the UK using a TTO method, for more information please see Szende, et al [5].

### EQ-5D-3L Survey

**Please indicate which statements best describe your own health state today.**

| **Mobility** | | **Coefficients** |
| --- | --- | --- |
| 1 | I have no problems in walking about | 0 |
| 2 | I have some problems in walking about | -0.069 |
| 3 | I am confined to bed | -0.314 |
| **Self-Care** | | |
| 1 | I have no problems with self-care | 0 |
| 2 | I have some problems washing or dressing myself | -0.104 |
| 3 | I am unable to wash or dress myself | -0.214 |
| **Usual Activities (e.g. work, study, housework, family or leisure activities)** | | |
| 1 | I have no problems with performing my usual activities | 0 |
| 2 | I have some problems with performing my usual activities | -0.036 |
| 3 | I am unable to perform my usual activities | -0.094 |
| **Pain/Discomfort** | | |
| 1 | I have no pain or discomfort | 0 |
| 2 | I have moderate pain or discomfort | -0.123 |
| 3 | I have extreme pain or discomfort | -0.386 |
| **Anxiety/Depression** | | |
| 1 | I am not anxious or depressed | 0 |
| 2 | I am moderately anxious or depressed | -0.071 |
| 3 | I am extremely anxious or depressed | -0.236 |

[5] Devlin N, Parkin D. Guidance to users of EQ-5D value sets. In: Szende A, Oppe M, Devlin N. EQ-5D Value Sets. 2007. EuroQol Group Monographs, vol 2. Springer, Dordrecht.
